# Antarctica challenges the new horizons in predictive, preventive, personalized medicine: preliminary results and attractive hypotheses for multi-disciplinary prospective studies in the Ukrainian “Akademik Vernadsky” station

**DOI:** 10.1186/s13167-016-0060-8

**Published:** 2016-05-31

**Authors:** Yevhen V. Moiseyenko, Viktor I. Sukhorukov, Georgiy Yu Pyshnov, Iryna M. Mankovska, Kateryna V. Rozova, Olena A. Miroshnychenko, Olena E. Kovalevska, Stefan-Arpad Y. Madjar, Rostyslav V. Bubnov, Anatoliy O. Gorbach, Kostiantyn M. Danylenko, Olga I. Moiseyenko

**Affiliations:** 1 National Antarctic Scientific Center of Ministry of Education of Ukraine, 16, Taras Shevchenko Boulevard, Kyiv, 01601 Ukraine; 2 Bogomoletz Institute of Physiology, National Academy of Sciences of Ukraine, 4, Bogomoletz str., Kyiv, 01024 Ukraine; 3 Institute of Neurology, Psychiatry and Narcology of the National Academy of Medical Sciences of Ukraine, 46, Akademika Pavlova str., Kharkiv, 61068 Ukraine; 4 Institute for Occupational Health of National Academy of Medical Sciences of Ukraine, Saksaganskogo str., 75, Kyiv, 01033 Ukraine; 5 Zhytomyr Ivan Franko State University, 40, Velyka Berdychivska Str., Zhytomyr, 10008 Ukraine; 6 G.S. Kostyuk Institute of Psychology of the National Academy of Pedagogical Sciences of Ukraine, 2, Pankivska str., Kyiv, 01033 Ukraine; 7 Budapest University of Technology and Economics, Budapest, Hungary; 8 Clinical Hospital ‘Pheophania’ of State Management of Affairs Department, 21, Zabolotny str., Kyiv, 03680 Ukraine; 9 Zabolotny Institute of Microbiology and Virology, National Academy of Sciences of Ukraine, 154, Zabolotny Str., Kyiv, 03680 Ukraine; 10 Ukrainian Academy of Informatics, Kyiv, Ukraine; 11 National Scientific Center ‘Mykola Strazhesko Institute of Cardiology’ of National Academy of Medical Sciences of Ukraine, 5, Narodnoho Opolchennya str., Kyiv, Ukraine

**Keywords:** Predictive preventive personalized medicine, Antarctica, Antarctic “Akademik Vernadsky” station, Physiology research, Disadaptation, “Antarctic syndrome”, Psychology, Pain, Anxiety, Photoperiodism, Desynchronosis, “Schumann resonance”, Infrasound, Climatic, Meteofactors, “Ozone hole”, Microbiota, “Sterile” environment, Vascular disregulation

## Abstract

**Background:**

Antarctica is a unique place to study the health condition under the influence of environmental factors on the organism in pure form. Since the very beginning of the scientific presence of Ukraine in the Antarctic, biomedical research has been developed for the monitoring of individual biomarkers of winterers and medical accompaniment in Antarctic expeditions.

*The aim* of the study was to analyze and discuss the retrospective data of long-term monitoring and observations in Ukrainian Antarctica station “Akademik Vernadsky,” providing multi-scale biomedical information with regard to conditions of a perfect isolation from technological and social influences and under extreme environmental factors.

**Methods:**

Medical and biological studies have been performed with the participation of all 20 Ukrainian wintering expeditions. We surveyed 200 males aged 20–60 years (mean age 37 years). Extensive medical examinations were carried out before the expedition, during the selection of candidates, and after returning, and particular functions were monitored during the entire stay in Antarctica. The medical records were analyzed to study the reaction of the human organism on phenomena like “Antarctic syndrome,” dysadaptation, anxiety, desynchronosis, photoperiodism, influence of climatic and meteofactors like “Schumann resonance,” infrasound, “ozone hole,” and “sterile” environment; important aspects of its role on human health were precisely studied and discussed.

**Results:**

The examinations showed the multi-level symptoms of the processes of dysregulation and dysadaptation, as functional tension in the sympathetic-adrenal system rights, especially during urgent adaptation to the Antarctic (1-month stay at the station) and, to a lesser extent, after returning from an expedition to Kyiv. At the initial, adaptation to the conditions of the Antarctic levels of urinary catecholamines (epinephrine, norepinephrine, dopamine, DOPA) increased compared with the start of the expedition (23.2 ± 4.3 and 53.3 ± 5 2 mmol/l, *p* < 0.001; 67.1 ± 12.3 and 138.3 ± 16.9 mmol/l, *p* < 0.01; 1749.6 ± 476.5 vs 7094.6 ± 918.3 mmol/l, *p* < 0.001; 129.6 ± 12.3 and 349.9 ± 40.6 mmol/l, *p* < 0.001, respectively). In the blood serum of 100 % of the expedition, we found an increase of oxidative stress markers—the level of TBARS increased by 41.2 %, i.e., the activation of free radical peroxidation. Thus, in 80 % of the participants, we observed a reduction in the activity of the SOD antiradical enzyme vs 58 % in the controls. Changes in brain electrical activity after a long stay at the Antarctic stations showed increasing delta rhythms, signs of CNS protective inhibition, likely due to hypoxia. We found changes in the concentrations of microelements (iron, copper, zinc, etc.) in the blood of winterers after the expedition. The polychrome-adaptive method of correcting the changes of the psycho-emotional state in a monochrome Antarctic environment was successfully applied.

**Conclusions:**

The preliminary results of the retrospective study and our own observations of the fundamental physiological mechanisms of the negative influence of extreme environmental factors on an organism in the absence of man-made origin factors allow the determination of many mechanisms of “pre-pathology” processes which promise to develop the pathogenetically based pro-active prevention methods for a number of common diseases to set prospective interdisciplinary research in predictive, preventive, and personalized medicine.

## Background

### Ukrainian Antarctica research meets the concept of predictive, preventive, and personalized medicine

Ukrainian science and, in particular, in Biomedicine in 2016 celebrates the 20^th^ anniversary of the research in the Antarctic. Ukraine had the opportunity to research activities in the Antarctic since 1996, when the British station “Faraday” was transferred to Ukraine and renamed with the name of the academician Volodymyr I. Vernadsky [1], the co-founder of geochemistry, biogeochemistry, and radiogeology, founder of the Ukrainian Academy of Sciences. The station works under the auspices of the Ministry of Education of Ukraine and is currently regulated by the Cabinet of Ministers of Ukraine Resolution number 1002 issued on November 3, 2010 [2]. The *Ukrainian Antarctic Journal* (UAJ) [3] is the official journal of the National Antarctic Scientific Center (NASC) of the Ministry of Education of Ukraine, established to disseminate results received in Ukrainian Antarctic Expeditions at the “Akademik Vernadsky” station and heard at biennial International Antarctic Conferences held by NASC and cover scientific fields: geological and geophysical, nuclear-physical, oceanographic, biological research, hydro-meteorological climatic research, geoinformation technologies, and medical and physiological research.

Since the very beginning of the scientific presence of Ukraine in the Antarctic, biomedical research direction has been developed for the monitoring of individual biomarkers of Antarctic winterers and medical accompaniment for Antarctic expeditions. Constant medical supervision (including the telemedicine options) of the state of health of Antarctic experts and new technology for monitoring the individual parameters of the functional state of the organism, despite the extreme conditions, allowed the maintenance of the health and performance of all the participants of 20 Ukrainian Antarctic expeditions (over 200). The main success of the Antarctic medicine is preserving the health of all members of the 20 expeditions, the creation of an effective system of medical and psychological selection of candidates, the development of health-monitoring methods for winterers at the Antarctic station, using telemedicine, and the introduction of innovative technologies for prevention, treatment, and rehabilitation. The main achievements of scientific developments in the field of medicine and physiology are to receive new information about human adaptation features in the extreme conditions of Antarctica; clarifying mechanisms of dysadaptative, desynchronosis, and “Antarctic syndrome” symptoms to determine the causes of stress; and problems of interpersonal relations in a small team, as well as a significant expansion of knowledge in the field of environmental physiology and extreme and preventive medicine. Special Antarctic environment for human is characterized by the absence of anthropogenic influences, limited color ambient gamma environment, the impact of changes of the electromagnetic spectrum, social deprivation, etc. That has allowed finding out the characteristic changes in the body’s functions and on this basis develop a number of completely new technology which increase the adaptive capacity of the body, correction of the psycho-physiological state, and rehabilitation to solve problems of *preventive medicine.*

Currently, action of the international coordination group for scientific and medical support of expeditions and organization of the international health systems in the Antarctic is under the auspices of the International Scientific Committee on Antarctic Research—Scientific Committee on Antarctic Research (SCAR) and the Council of Managers of National Antarctic Programs (COMNAP) [3]. This work involved 24 COMNAP countries (including Ukraine), which have national Antarctic programs and experience in medical support of expeditions. Medical standards and the volume of medical examinations of candidates to participate in an expedition have many differences among participating countries that depend on many factors, like the conditions of the expedition, geographical location of the station, and the duration of stay, as well as on national (ethnical), economical, and legislation factors.

The results of a special survey by COMNAP allowed to summarize the status of work on the medical examination of candidates to participate in the Antarctic expeditions in different countries. The general and leading position in all countries is that the participants of Antarctic expeditions must have good health, good physical development, and the body’s ability to adapt to the extreme Antarctic conditions. Therefore, in all countries, rigorous medical screening is organized for the candidates for Antarctic expeditions.

The unique challenges posed by the Antarctic environment to the human body have been previously well described [4–9], and the differences of morbidity in Antarctica were reported [10–12]. Thus, cold injury is uncommon in Antarctica. Despite this, it warrants a continued high profile as under most circumstances it may be regarded as an entirely preventable occurrence [10]. Injuries continue to be the most common cause of morbidity in the 27th Indian Scientific Expedition to Antarctica (ISEA). Nutritional deficiencies and cold-related injuries are relatively less common [11]. In the Chinese Antarctic Research Expedition (CHINARE), traumatic injury remained the most common medical condition during the Chinese Antarctic expedition. Preventive care is the most cost-effective strategy of medical care [12]. The team doctors were recommended be well trained to manage injuries and diseases with limited medical resources.

The concept of predictive, preventive, personalized medicine (PPPM) is developing in many aspects of human health [13–18]. Antarctica being a unique place considered for monitoring the health condition under the influence of environmental factors on the organism in pure form poses new challenges for predictive, preventive, personalized medicine.

*The objectives of the study were* to analyze and discuss the retrospective data of long-term monitoring and the observations of the dynamics of the human body function in environmental conditions in the Ukrainian Antarctica station “Akademik Vernadsky” (simulating the conditions of a perfect isolation from technological and social influences) and to analyze the biomedical information with regard to extreme environmental factors to determine the mechanisms of “pre-pathology” process that allow to develop pathogenetically based pro-active prevention methods for a number of common diseases which may be promising for interdisciplinary research in predictive, preventive, and personalized medicine.

## Methods

### Ukrainian Antarctic scientific “Akademik Vernadsky” station

**Fig. 1:**
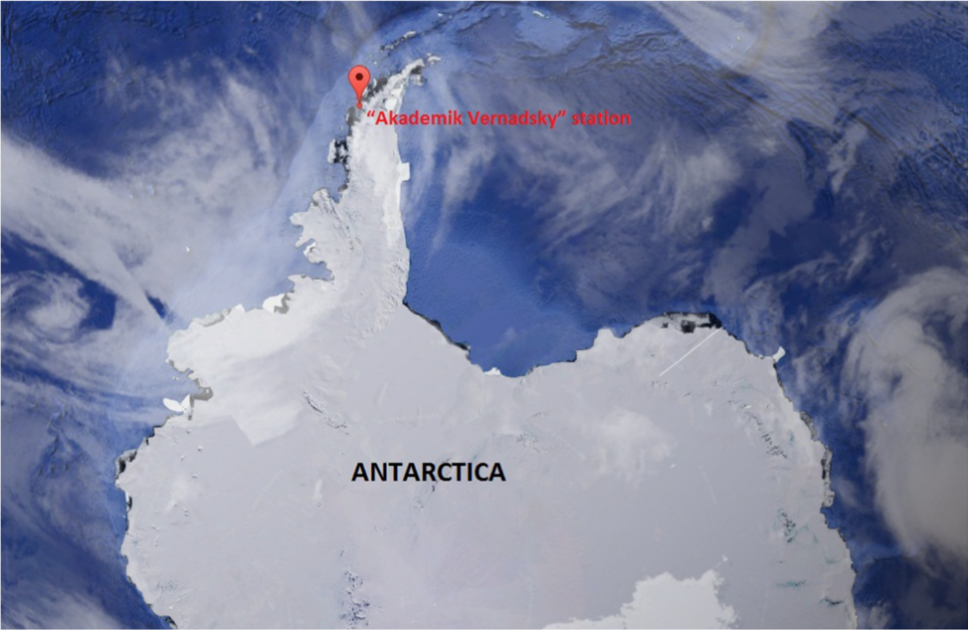
The geographical location of the scientific stations in Antarctica

The geographical position of the Ukrainian Antarctic scientific “Akademik Vernadsky” station is defined by the coordinates 65° 151 south and 64° 161 west longitude. It is located on the island of the Galindez archipelago near the Argentine Islands (Strait 7 km) of the Antarctic Peninsula (see the map on Fig. 1). The island location of the station is characterized by a limited area (the island diameter of about 1 km), and two thirds of the island is covered with the spherical surface of the glacier and the rest of the territory—the rocks of volcanic origin—making it difficult for pedestrian movement and making it impossible to land aircraft. Nearby islands are located within a radius of 35 km, to which members of the expedition in the summer may be reached by boat, and in the winter (in the case of formation of safe ice) on the ice surface, which is always accompanied by a certain risk. Regional climatic conditions make possible the maritime transport approach in the summer (February–March), when changing the station crew. The region of the station is characteristic of high latitudes photoperiodicity with long winter nights and extra-long summer days.

All time zones were shifted to 6 h to the west of Ukraine, and the seasons are reversed. Global helio-geophysical and meteorological processes in the region of the station each year, within 3 months (August, September, October), naturally lead to a reduction in the thickness of the ozone layer in the atmosphere (the “ozone hole”). The thickness of the ozone layer is reduced to 150 Dobson units (compared to the standard 325 units in Ukraine), which never happens in Ukraine. This phenomenon leads to a significant increase of solar radiation in the ultraviolet range. The conditions of the coastal Antarctic, where the station is located, has increased activity of weather and helio-geophysical phenomena. Weather conditions can change dramatically even within a day. The cyclonic activity in the region and the number of stations with increased cyclones per year exceed the number in Ukraine in two times. In addition, their duration in the Antarctic is much more powerful and higher than that registered in Ukraine. Subzero temperatures in the winter rarely exceed 300 °C and in summer during a very short period (only during the day) can rise over 0 °C. The average annual temperature in the region of the station is 40 °C, compared to +90 °C in Ukraine. At the same time, throughout the year, at the station, there is the number of days with temperatures below zero value, which corresponds approximately to the number of days with temperatures above zero in Ukraine (270 and 280 days, respectively). Precipitation in the form of snow and rain come in almost every day. In winter, there are snow drifts up to 2–3 m. However, total rainfall for the year is not higher than in Ukraine. The station area is always with a humidity increased to 80–90 %. The sun appears for about 35 days during the year. The duration of sunshine in Antarctica during the year is almost four times lower compared with that of Ukraine. Strong hurricane winds and sudden changes in barometric pressure (40 mmHg or more per day), which are stored for a long time, lead to the fluctuation of oxygen in the ambient air and reduces its partial pressure greater than 10 mmHg. In addition, infrasound test load (in the band 6–7 Hz) of the Antarctic station members of the expedition, which exceeds the background values in Ukraine, almost doubled. The direction of the field lines of the magnetic field of the Earth in Antarctica is reversed, and helio-geomagnetic phenomenon is more pronounced, which is fixed by magnetometric equipment. Landscape environment of the station is characterized by monotony, monochromeity (black and white), natural purity, and “sterile” environment that has not experienced significant influence of the harmful factors of anthropogenic origin.

### Living conditions at the Antarctic station

**Fig. 2:**
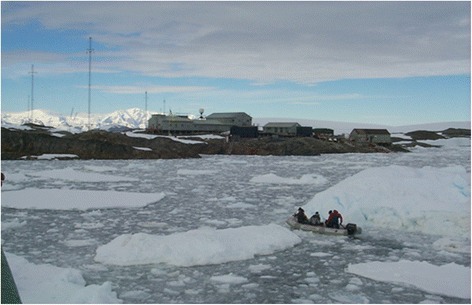
General view of the Ukrainian “Akademik Vernadsky” Antarctic station and the area around

The station is a well-equipped research laboratory with constant electricity, modern electronic and home appliances, and residential and ancillary facilities, which are all necessary for human existence, with reliable technical life-support systems. However, members of the expedition (12–15 persons) (up to 13 months) are removed for a long time from familiar surroundings of the civilized world, working in a limited area in a small team environment under the conditions of social and psychological isolation, sensory deprivation, and sexual abstinence. Territorial restrictions and the long Antarctic winter create the conditions for forced communication among a small team of members of the expedition and the development of a certain state of inactivity during the winter. Living conditions at the plant excludes the possibility of hypothermia, starvation, lack of water, and electricity. Environment in the station area is free of man-made origins of pollution and in neighborhoods formed a certain vicious bacterial microclimate without exogenous exchange outside of human viral and bacterial flora. Sanitation characteristics of housing and work premises meet the existing standards in Ukraine. The general view of the area around the station is presented on Fig. 2.

Meals were served three times a day; the simple dishes and drinks, which do not require complex preparation technology, are served for breakfast, lunch, and dinner and are prepared by a qualified chef. For all members, there was an option for extra portion. In calculating the daily calories of the food for the participants of the expedition, the standards used for professional extreme activities (submariners) were used. Water is supplied by desalination equipment. For cooking, demineralized water was mainly used, and there is a shortage of dairy products. At the station, there are a medical and laboratory rooms that are equipped with modern diagnostic and therapeutic agents and pharmacological for a wide purpose. The station has a sports hall with the necessary equipment for exercises. The physical activity of members of the expedition outside the station premises is dependent on the time of year. In winter, the expedition members periodically perform works on clearing the snow drifts. The main exercise of the station crew is in the summer, when tourists start arriving on numerous cruise ships which operate seasonally, groups of researchers carry out repairs and unloading operations, and weather conditions allow carrying out of research far beyond the station using technical vehicles (motor boats). The station crew usually consists of six persons of support staff (a medical doctor, a cook, an electrician, technician, mechanic, radio operator) and six to eight researchers (physics, geology, meteorology, biology). Communication of station crew members with the outside world was conducted via satellite telecommunications.

### Diagnostics and physiology research for preventive medicine

Medical and biological studies have been performed with the participation of all 20 Ukrainian wintering expeditions.

We surveyed males aged 20–60 years (mean age 37 years, the crews of the station). All medical examinations were carried out before the expedition, during the selection of candidates on medical criteria. During the expedition to the Antarctic station, the performance of individual functions of the body was monitored during the entire stay in Antarctica. After returning from the expedition, medical examinations were carried out in full, according to a similarly launched protocol. Complex medical examination for the selection of winterers included the following:

- *Clinical diagnostics* specialists consultation (internist, surgeon, neurologist, psychiatrist, dermatologist, ENT doctor, dentist);
- *Diagnostic techniques* (ultrasound, X-ray, electrocardiography (ECG), magnetocardiography, zonal rheography, body plethysmography, laboratory tests of blood and urine), measuring the concentrations of microelements (iron, copper and zinc) in the blood serum;
- *Functional diagnosis* using the bicycle ergometry method (determination of physical exercise tolerance), hypoxic training technique (sessions breathing air with reduced oxygen content (in the range 12–18 %) under the control of blood hemoglobin oxygen saturation using oximetry), the method of determining the level of stability with statokinetic using stabilography and encephalography;
- *Psychological and psycho-physiological diagnostic examination* with individual features electric brain biorhythm registration by conventional electroencephaolography (EEG) and using the package of psychological tests to determine the characteristics of the person (emotional stability, fatigue resistance, the type of higher nervous activity indicators sensorimotor reactions, attention, reaction to an object that is moving, speed switching of attention, visual and auditory memory, orientation in an enclosed space);
- *Genetic diagnosis*, which included the micronucleus test (qualitative and quantitative characterization of micronuclei and nuclear anomalies in the *buccal cells*) and genotyping oxygen-dependent domain HIF-1 (hypoxia-inducible factor, which is a key transcription factor that contributes to the regulation of target gene expression in and hypoxic stress conditions) for the presence of allelic polymorphism of HIF-1 gene. HIF is under the control of the expression of several genes that control the synthesis of erythropoietin, vascular endothelial growth factor, enzymes of glycolysis, ceruloplasmin, nitric oxide, etc. All of these proteins promote the organism adaptation to hypoxia and other extreme influences;
- *Morpho-functional studies of the organ of vision* included the following: evaluation of complaints about the quality of (by National Eye Institute (USA) Visual Function Questionnaire-25 -NEI VFQ-25) general eye exam using routine methods—biomicroscopy, direct and inverse ophthalmo-chromoscopy, gonioscopy, measuring visual acuity on Sivtsev’s tables, and intraocular pressure with the introduction pneumotonometry. We also conducted in-depth study of the optical density of the cornea and lens densitometer method, corneal thickness, and its state of the endothelium with *cornealpachymetry* and endothelial microscope, morphological status of the optic nerve and retinal nerve fiber layer using *Heidelberg retinal tomography* (HRT) of the morphological status of the retina in the macular area—the method of *optical coherence tomography* (OCT), and its sensitivity was studied using quantitative perimetry.

We studied the morphological and functional characteristics of the mitochondria of leukocytes using electron microscopy.

### Health monitoring at the Antarctic station

*Monitoring* of winterers’ functional systems at the Antarctic station was carried out using electrocardiography (ECG) and electroencephaolography (EEG) techniques, spirometry, rhythmocardiography, measuring blood pressure, temperature, and methods of psycho-physiological testing via software packages (forecast system).

On the Antarctic station, all studies were done by medical doctors daily (blood pressure, pulse, temperature), monthly (bicycle ergometry, electrocardiography, electroencephalography, spirography, rhythmocardiography), and quarterly (psychological testing, clinical and biochemical blood tests, densitometry).

EEG registration was carried out by the usual method using the automated complex, which consists of an electroencephalograph, a laboratory, and a computer interface. The working program was “EEG Mapping 3.” EEG potentials were recorded monopolarly in the frontal (F3, F4, F7, F8), central (C3, C4), parietal (P3, P4), temporal (T3, T4, T5, T6), and occipital (O1, O2) areas of the brain according to the “10-20” system. Reference electrodes were combined together except for the electrode used in each lead. Filter cutoff frequency of high and low frequencies were, respectively, 1.5 and 35.0 Hz, the frequency of the signal digitizing—250 c-1. EEG signals were processed using a fast Fourier transform using the smoothing Blackman method. Epoch analysis for FFTs was 2.56 s. The study included the registration of the EEG with closed and open eyes, as well as visual and emotional stimulation of neutral, positive, and negative modality according to the International system of Affective Picture System (IAPS) stimuli which imposed 30 stimuli for each modality. The duration of each stimulus was 1000 ms, and interstimulus period was from 3000 to 4000 ms. We determined the averaging values of the following ranges: θ-rhythm (4–8 Hz), α-rhythm (8–13 Hz), β1-rhythm (16–20 Hz), and β2-rhythm (21–30 Hz). We analyzed the average value of the power (V/Hz) EEG rhythmic components in the background recording, as well as during EEG recording with eyes open and during the presentation of visual stimuli. In addition, the expected rate of EEG desynchronization was relative to baseline values during the functional tests. Desynchronization factor analyzed on EEG rhythms for each of the samples was calculated from the formula (1):

$$ \left(A/B\right)\ *\ 100\ \%\ (1), $$

where *A*—indicators of the power of EEG rhythms during the functional test, in—power values of the background EEG. The data were processed by standard methods of statistical variation. The EEG power averages (V/Hz) and the standard deviation of the averages were taken for analysis.

### Information technologies

For operational analysis of the survey results, we used telemedicine transmission of information technologies in Ukraine. To correct the psychological dysfunctions, we used the technique of “Polychrome Bio-regulation” as described in [19].

After returning from the expedition, we used complex surveys similar as at the initial stage of medical examination.

### Statistics

The level of statistical probability of intergroup differences was calculated using the ANOVA criterion. The mathematical processing of the data was performed using STATISTICA 6.0 software.

### Ethics

The current study and all measurements, monitoring, diagnostic, and therapeutic procedures performed at the Antarctic station and related to our report were approved by the National Antarctic Scientific Center of Ministry of Education of Ukraine, and the results are allowed to be published by decisions of the Scientific and Technical Council of the “State Target Scientific and Technical program of research in Antarctica for 2011-2020” (STC “Antarctica”)—protocol N 3 was issued on December 23, 2015.

## Results and discussion

At the Ukrainian Antarctic station, the medical escort crew for 20 years was based on the principles of preventive medicine: an individual assessment of the risk of possible occurrence of disease (genetics, diagnosis, assessment of psychophysical condition) to develop anticipation strategies, regular monitoring of the state of the marker indicators that reflect the current state bodies and winterer’s health systems, and use of appropriate measures to prevent the development of critical situations in health [20–36].

The frequency and structure of diseases at Vernadsky station during 10 expeditions is presented on Table 1 based on the data of requests for medical aid.

**Table 1:** The structure of diseases at the “Akademik Vernadsky” station (data of requests for medical aid), %

Diseases	Expeditions								
	1	2	3	4	5	6	7	8	9
Surgical and trauma	8.3	52.1	54	15.8	10.8	41.8	27.7	22.6	17.2
Internal and neurological	43.8	22.0	32.6	52.6	58.8	24.6	37	45.9	70
Dermatological	10.4	1.3	2.9		6.5	20.9	22.2	8.8	5.5
Ophthalmological		8.2	8.6	5.3	10.8	0.9	6.5	5.2	2.8
Dental	37.5	15.1	1.9	22.1	13.1	2.7	3.7	8.0	2.8
Otolaryngological	–	–	–	2.1	–	7.3	1.9	8.8	1.15
Urological	–	1.3	–	2.1	–	1.8	0.9	0.7	0.55

Preventive medicine is most closely connected with the study of physiological and pathophysiological mechanisms, which elucidation significantly increases the efficiency of measures preventing the development of diseases and disorders of the body.

However, to date, mechanisms of many diseases and pathologies, including the widespread state of chronic stress, unknown etiology headaches (migraine); affective, depressive, and neurotic states; meteodependence; and others, are not fully clarified.

In order to study the mechanisms of development, this type of pathology requires special conditions that are practically impossible to create in an environment of modern civilization, which has brought a huge amount of negative effects on human artificial origin.

At the Antarctic station, a unique possibility exists to monitor the health conditions under the influence of environmental factors on the organism in a relatively undisturbed form, with limited interferences from other environmental factors. For example, it is known that the results of medical observation in Antarctic conditions, with a small team, of social deprivation have long been used in predictive calculations for planning *long-term space flights*. Professional activities in Antarctica are associated with isolation in a small team, social and sexual deprivation, and unusual activity of the regional natural factors that could adversely affect its psycho-physiological status. It is also known that a change in the biological rhythm of the normal functioning of the human body systems in its activities in the Antarctic is reflected not only on health but also on his ability to work. In the body, there are violations on the system, organ, and cellular levels able to influence or encourage the development of, for example, hypoxic conditions and psycho-emotional disorders. Long-term medical surveillance in the “Akademik Vernadsky” Antarctic station allowed to identify traditional wintering diseases and apply therapeutic and preventive measures to organize optimal conditions for the existing medical support system. However, cases of violations of adaptation and symptom manifestation of “Antarctic syndrome” continue to take place and constitute the central link among the unresolved issues of adaptation [27, 28].

In recent years, biomedical research at the station “Akademik Vernadsky” did a major contribution to the information base, which suggests new strides in addressing the life sciences and to identify promising avenues for further scientific research. During to the examination of medical and physiological conditions, we identified the distinct seasonal periods of occurrence psycho-physiological disorders in winterers with prolonged change in the psychological characteristics of the person. Such disorders can lead to a state of chronic stress and provoke the emergence and development of conflicts. Results of winterers’ EEG analysis showed signs of irregularities in the structure of biorhythmic organization, especially those parts of the brain that are related to the psycho-emotional sphere [24, 25]. The features of rearrangements cerebral biorhythms of winterers after a long stay in the extreme conditions of the Antarctic and the visual sensory deprivation were the gaining power θ-, β1-, and β2-EEG rhythms, mainly from the projections of the central and frontal areas of the cerebral cortex (compared with the original data by 25.6, 24.6, 33.6, respectively). EEG rhythms in the medium- and high-frequency range (α- and β-rhythms) were recorded while maintaining the visual sensory, statistically significantly increased, mainly in leads from the projections of the central regions of the cerebral cortex. It is known that the increased activity of θ- and β-rhythm is related to the level of anxiety, stress state, and the development of various autonomic changes in the human body. In addition, the performance gain β2-rhythm may be associated with a level of emotional background. During hibernation, physiological changes can be triggered by members of the expedition factors’ biorhythmic nature. Indeed, at the Antarctic station, the biorhythm of the usual psycho-physiological functions of winterers was reconstructed.

**Fig. 3:**
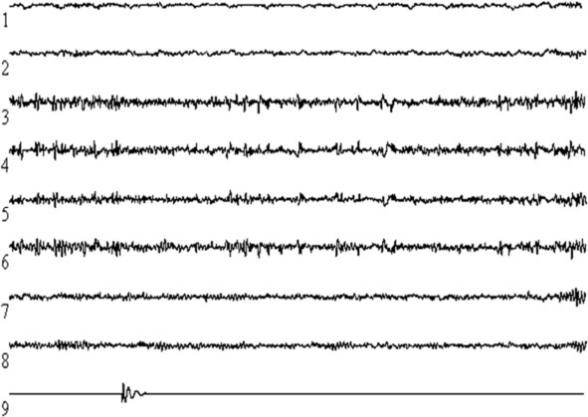
The reaction of the electrical activity of the human brain to electromagnetic stimulus. (*1*) frontal drainage of the left hemisphere (SF-Pa), (*2*) frontal withdrawal right hemisphere (DF-Pa), (*3*) parietal EEG—left hemisphere (SPa-Pp), (*4*) parietal EEG—right hemisphere (DPa-Pp), (*5*) occipital EEG of the left hemisphere (SPp-O), (*6*) occipital EEG of the right hemisphere (DPp-O), (*7*) temporal EEG of the left hemisphere (SO-T), (*8*) temporal EEG of the right hemisphere (DO-T), (*9*) artificial electromagnetic pulse; calibration 100 mV/15 s

We revealed that the end of winter and after returning from the expedition, there was a significant decrease in the amplitude of high rhythms (beta and gamma activity by 2.4 and 1.2 %, respectively), the amplitude of the alpha rhythm decreased by 3.8 %, and delta and theta rhythms were increased by 0.4 and 7.1 %, respectively (Fig. 3).

Changes in brain electrical activity participants of Antarctic expedition after a long stay at the Antarctic stations were mainly represented by an increasing power of the delta range that can be a manifestation of energy change and development processes of protective inhibition of the central neural system (CNS), likely due to the impact of certain mechanisms of hypoxic origin.

It is known that a stable structure of biorhythmic (circadian) psycho-physiological functions of the human is an indicator of the effectiveness of adaptation. Thus, the speed and structure of these individual “adaptative alterations” have certain features that may affect the quality of adaptation. Thus, human adaptation in Antarctic conditions under the constant change of biorhythms and, as demonstrated by the data in Table 2, requires significant functional strain of the organism as a result of seasonal invert normal circadian architectonic psycho-physiological functions. In addition, the adaptation process develops gradually, stage by stage, with a minimum efficiency at the beginning, some increase in efficiency in the middle of winter, and its decrease at the end of winter. Increased tension of functional systems is reflected in the psycho-emotional state, as evidenced by the dynamics of the level of anxiety within the team structure of winterers (Table 3).

**Table 2:** Dynamics of the number of winterers with appropriately structured architectonic circadian rhythm psycho-physiological parameters (%)

Parameter	Autumn	Winter	Spring	Summer
Self-esteem	18.7	21.2	27.8	16.7
Activity	12.5	42.2	16.7	16.7
Mood	12.5	26.3	33.3	11.1
The speed of information processing	28.6	26.1	45.2	38.1

**Table 3:** The dynamics of the level of anxiety indicators among winterers (%)

The level of anxiety	Antarctic seasons			
	Autumn	Winter	Spring	Summer
Normal	14	7	4	14
Above average	36	32	50	14
Below the average	36	36	42	58
High	14	25	4	7
Low	-	-	-	7

Thus, polymorphic restructuring of human psycho-physiological functions under the influence of a complex emergency in the Antarctic factors complicates the adaptation and leads to increased tension, and disorder and *desynchronosis* elongated stress state can modify numerous compensatory-adaptive mechanisms in the course of adaptation to winter conditions. Increased levels of tensions and signs of stress confirmed the increased secretion of catecholamines in winterers. At the same time, against the background of the identified general physiological rules, the adaptation period for each winterer demonstrates some individual features.

At the initial adaptation to the conditions of the Antarctic levels of urinary catecholamines (epinephrine, norepinephrine, dopamine, DOPA) increased compared with start of the expedition (23.2 ± 4.3 and 53.3 ± 5 2 mmol/l, *p* < 0.001; 67.1 ± 12.3 and 138.3 ± 16.9 mmol/l, *p* < 0.01; 1749.6 ± 476.5 vs 7094.6 ± 918.3 mmol/l, *p* < 0.001; 129.6 ± 12.3 and 349.9 ± 40.6 mmol/l, *p*< 0.001, respectively). After returning from the expedition, the levels of the mentioned catecholamines in daily urine were at the initial state, and some were slightly elevated (respectively for epinephrine, norepinephrine, dopamine, DOPA: 35.1 ± 7.2 mmol/l, *p* < 0.2; 91.9 ± 25.9 mmol/l, *p* < 0.5; 2122.3 ± 860.3 mmol/l, *p* > 0.5; 246.7 ± 65.9 mmol/l, *p* < 0.1). The level of 17-ACS in urine after returning from the wintering expedition was characterized by a tendency to increase from 0.25 ± 0.04 u.o. (in baseline) vs 0.34 ± 0.05 u.o. (0.05 < *p* < 0.2). Thus, studies showed for the availability processes dysregulation and functional tension in the sympathetic-adrenal system rights, especially during urgent adaptation to the Antarctic (1-month stay at the station) and, to a lesser extent, after returning from an expedition to Kyiv.

It was more complicated to determine the genesis of the functional state of the organism’s oxygen transportation under prolonged exposure to the Antarctic stations in the region which location hypoxia apparently absent. In late winter, the body members of the expedition revealed activation of free radical peroxidation of lipid peroxidation branching process, as evidenced by the accumulation in the blood serum of its end products—low molecular markers of oxidative stress, such as compounds that react with thiobarbituric acid reactive substances (TBARS). The results of biochemical studies of the blood serum of 100 % of the expedition had exceeded the baseline levels of TBARS (at average 41.2 %), i.e., the activation of free radical peroxidation processes. Thus, 80 % of the patients showed a reduction in the activity of superoxide dismutase (SOD) enzyme antiradical with a maximum deviation of control at 58 %.

After a long Antarctic expedition, individual winterers tend to reduce the openness of character, and features of a total state of euphoria were found. This situation is characterized by deep emotional expressiveness and subjective experiences inspired by the long stay in the extreme conditions of the Antarctic expedition. Dynamics of indicators rhythmocardiography of winterers testified modifying the balance of the autonomic regulation. After the expedition, in winterers, we revealed significant changes in the system of multi-level regulatory function statokinetic support sustainability. The individual encephalography data characteristic variations were showed to might depend on the psycho-emotional origin. After wintering, typical morphological and functional changes of mitochondria in the blood system were revealed. The results of ultramicroscopic studies of mitochondria of leukocytes of winterers indicate that once a long stay in the Antarctic accompanied by development signs of mitochondrial dysfunction, and if hibernation is repeated three to four times in winterers that were observed as the development of certain adaptive reactions on the part of the mitochondrial apparatus of leukocytes, but a further increase in the number of stays in Antarctica can cause pathological changes in the ultrastructure of the mitochondria with signs of destruction of organelles, which may occur due to pronounced *oxidative stress* [26]. Signs of mitochondrial dysfunction and surge clear after hypoxic stimulation of winterers. At the same time, the mRNA expression levels of the HIF-1α gene peripheral blood leukocytes were increased in winterers and are closely correlated with indicators of the implementation of the functional reserves of the respiratory and circulatory systems [30–36]. Thus, the findings confirm the presence in winterers’ features of overvoltage functional systems. The dynamics of such changes can have individual variations during hibernation. This may be related to the personal characteristics of winterers and with individual susceptibility to the effects of a complex of extreme Antarctic factors.

There is a reason to believe that the process of adaptation to Antarctic conditions has a phase character. Conventionally, in the process, we can highlight the acute phase of adaptation, functional stress, relative stabilization, and depression. The acute phase of adaptation (Antarctic autumn) is characterized by active processes’ biorhythmic nature and confirmed the normal circadian disorders’ architectonic psycho-physiological functions, as well as body temperature, which is the daily dynamics of the indirect evidence of the violation of the rhythm of melatonin secretion. Changes in the biological clock may be accompanied by increased stress of the central mechanisms of the regulation of functional systems, which is manifested in the activation of cerebral electrogenesis, as well as changes in self-esteem and individual psycho-physiological qualities. Phase voltage function (Antarctic winter) is the result of complex influence biorhythmological factors, deprivation and physical inactivity and is characterized by the end of the formation of interpersonal relations in the team, the individual polymorphic manifestations of “Antarctic syndrome,” and characteristic changes in the ratio of power rhythms of the electrical activity in the brain. For example, in an awake winterer, the brain biorhythms were inherent for a sleeping man. In the phase of relative stability (Antarctic spring), in a group formed by interpersonal relationships, the number of *desynchronosis* manifestations is reduced, optimized EEG rhythms. The final phase of depression (Antarctic summer) is characterized by increased levels of anxiety, irritability, emotional instability, increase in the proportion of low-frequency EEG rhythms, and *desynchronosis* disorders [24, 25].

According to the previous expeditions’ experience and research results, the most important in disease prevention in wintering are the following: medical screening, appropriate use of therapeutic and preventive tools, special education, and training station operators. This is due to adaptation to extreme conditions of the Antarctic environment and the specifics of the professional activity of the person in the expedition to the “Akademik Vernadsky” station. Similar problems emerge in the crews of all the scientific stations in the Antarctic, the Arctic, and in other conditions where the work is done in isolation from the traditional society under the influence of the complex body of extreme environmental factors. Therefore, the results of the research on the analysis of the Ukrainian Antarctic station can be taken into account improve the efficacy of international activity for the long and safe stay of humans in the Antarctic.

### “Schumann resonance”

We studied the biological response of the human organism under the impact of global electromagnetic radiation. Particular attention was focused on the subrange of the global radiation energy, since the frequency characteristics of the so-called Schumann resonance coincide with the frequencies of the main electrical rhythms of the brain activity of a person, which can cause the timing to effect of dysregulation and dysadaptation disorders [20].

Such range of radiant energy outside Antarctica can be generated by sources of man-made origin; however, the study of the biological response to such stimulation is still at an early stage. It has been shown that even brief exposure to the natural power of the electromagnetic pulse leads to generalized changes in the cerebral biorhythms in humans. At the same time, the structure of the paroxysmal activity (alpha and theta range) is monomorphic, but in the paroxysms, the pointed wave is included, which is an evidence of short-term generalized activation reaction. On the other hand, it was found that the previous CNS stimulation by pulse low-frequency electromagnetic radiation through a certain period of exposure leads to weakening of the biorhythmic reaction of the human brain. Therefore, in order to increase the health stability of the wintering personnel to these negative influences, a special training technique specific to the action of low-frequency radiation has been developed. The method is based on a method of *preventing* the negative impact of low-frequency electromagnetic field on the psycho-physiological state of a person through the use of a remote session dosage range of artificial stimulation of the magnetic field, which contributes to the activation of non-specific mechanisms of human adaptation to extreme conditions. Further study of the synchronized reactions’ biorhythmic activity of the brain under the influence of artificial electromagnetic stimulation in clinical practice has allowed establishment of the positive effects of such stimulation in the treatment of patients with neurological diseases. The result is a new treatment technology that was implemented into clinical practice.

### Subsonic (infrasound) background

The highest values of the average level in the region of infrasound station in Antarctica in the range of 6–7 Hz observed from July to September, the lowest—in March. However, there are periods of short-term exceedances of the threshold of sensitivity, even in a relatively quiet time. At the background level at an average of 60 dB, we observed bursts that exceed the threshold of 90 dB. In such conditions, for winterers at the station (especially in the adaptation disorders), it may deteriorate performance and psychological state [36].

In this regard, the study of the reaction of the organism on the wintering effect of infrasound is very important. In order to clarify the nature of the impact of infrasound oscillations on the circulatory system function, the data results of simultaneous monitoring during the hibernation year, twice a day, and the measured blood pressure (BP) and heart rate (HR) for each of the wintering personnel (*n* = 11) are analyzed. For the meteorological parameters of the atmospheric pressure, measured at 5-min intervals, up to two values were averaged per day. The same procedures were subjected to infrasound measurement value (initial discontinuity 20 Hz). Cluster analysis based on the values of heart rate, systolic, and diastolic blood pressure was done and allowed to divide the set of normal wintering into two groups, under the peculiarities of the represented circulation. To determine the exposure to infrasound on winterers, we held a multi-regression analysis, whereby we found that a cluster of “0” is the correlation coefficient over 0.51. This result indicates a significant effect on blood pressure, atmospheric pressure drops, and background levels of infrasound. It is shown that the group of zero cluster generally reacts strongly to meteorological factors, which is the manifestation including infrasound (for example, a decrease in atmospheric pressure often increases the wind speed in the surface layer, and the wind, just like a powerful infrasound generator). Almost always, there was a definite relationship: under an increasing level of infrasound, we registered an increase of the coherence of the physiological data series. In addition, for the time interval (from April 1 to October 1), there was a steady increase in the coherence of the whole index wintering cohort that could be associated with rearrangements of adaptive mechanisms, development of *desynchronosis* displayed at the changed photoperiodicity, etc. Visual comparison charts for the coherence of the physiological series with graphs of the dynamics of infrasound resulted in the following pattern: with an increase in the level of infrasound was a slight increase in the level of coherence of physiological data, which confirms the link between physiological parameters and external factors.

### Meteofactors

Research on the influence of meteorological factors on the human body have discovered that this heart function depends on changes in atmospheric pressure and ambient air humidity. The rapid drop in atmospheric pressure (due to the high humidity) is always accompanied by a decrease in the oxygen partial pressure indicating the hypoxic nature of the growth mechanisms of the heart with the sharp drops in barometric pressure (characteristic for Antarctic conditions).

The results of this analysis showed the close correlation between the parameters of barometric pressure and humidity of the outside air and the heart function as a pump (myocardial performance index, minute volume, cardiac index (CI)) and systemic vascular resistance (SVR). Moreover, with respect to that of ISM, minute volume and CI correlation of changes in barometric pressure and humidity was negative, and the changes of SVR positively correlated with meteorological parameters. Correlation analysis showed (Table 4) the close relationship of barometric pressure parameters of ECG parameters that have some kind of interval R-R and complex QRS. Thus, humidity had a slightly lower correlation of dependence of the specified parameters of the ECG interval including the P-Q. Besides, a quite strong direct correlation between parameters of ozone levels and coefficient of variation (K_i_) and QRS intervals on the ECG was found.

**Table 4:** Correlation of geophysical and ECG parameters in winterers (Antarctic autumn)

Parameter	R-R	P-Q	QRS	Q-T	SP	I	II	III	IV	V
R-R, ms	1									
P-Q, ms	0.73	1								
QRS, ms	0.38	−0.10	1							
Q-T, ms	−0.71	−0.15	−0.28	1						
SP (pre-excitation syndrome), %	0.88	0.84	0.44	−0.32	1					
CI, u.o.	0.87	0.89	0.34	−0.29	0.99	1				
К_i_, u.о.; I	−0.22	−0.30	0.70	0.47	0.09	0.02	1			
Ozone level, II	0.09	−0.26	0.94	0.03	0.26	0.16	0.89	1		
Atmospheric pressure, hPa; III	−0.91	−0.57	−0.70	0.57	−0.90	−0.86	−0.16	−0.47	1	
Humidity, %; IV	−0.70	−0.89	0.37	00.38	−0.61	−0.68	0.69	0.59	0.39	1

On the other hand, a direct correlation between total vascular resistance indices and atmospheric pressure changes suggest a possible stimulation of vasoconstrictor mechanisms in the fall of barometric pressure, which is also a sign of hypoxia. There is clear likelihood that the effect of hypoxia is associated with molecular-genetic mechanisms of adaptive reaction Antarctic wintering. It was found that in patients with heterozygous genotype HIF-1a (C/T genotype) there are more favorable conditions for the development of dysadaptation violations, especially by the mechanisms of regulation of the body oxygen regime [37, 38]. At the same time, in heterozygous wintering in forwarding activities in Antarctica, functional stresses in the circulatory system were more expressed and promoted the speedy exhaustion of functional reserves. Hypoxia, which occurs as a result of grueling physical work, in patients with heterozygous genotype HIF-1a hemodynamic efficiency level of oxygen regime regulating the body was decreasing, which might be due the individual reactions, which are regulated by mechanisms at the molecular and genetic level.

The knowledge of the molecular and genetic aspects of the origin of adaptation and disadaptation rearrangements opens the possibility of developing genetic and pharmacological methods, which are designed to enhance, block, and modify the adaptive response of the body. It is shown that in order to increase the adaptive capacity of the body’s members, hypoxic training for Antarctic expeditions can be applied.

In the study of the influence of meteorological factors using mathematical modeling techniques to analyze the results of simultaneous monitoring of biomedical and meteorological parameters in the conditions of a long wintering Ukrainian Antarctic expedition at “Akademik Vernadsky” station, we used a database of observations of atmospheric pressure changes and physiological parameters of personnel during the Antarctic expedition for the modeling. The possibility of using mathematical models (discrete models of dynamical systems) to determine the stability of the regulatory influences of the autonomic nervous system to the atmospheric pressure change in the circumstances of Antarctica was considered. The results obtained a model-mathematical analysis which allowed evaluation of the sensitivity of heart rhythm regulation mechanisms to the barometric pressure changes during the Antarctic winter.

### Blood microelements—markers of stress and adaptation

Measuring the concentrations of microelements (iron, copper, and zinc) in the blood of winterers (by atomic absorption spectrophotometry in flames after wet mineralization of samples) after the expedition provides the evidence on a shift in the structure and trace-element composition in the blood serum during the expedition. We found that the microelement content in the blood of members during the wintering expedition (0.26 ± 0.03 mg/l) and after (0.24 ± 0.03 mg/l) are appropriate to the metal content in the control (0.27 ± 0.03 mg/l). Iron content in the serum of the wintering expedition to (1.65 ± 0.19 mg/l) was significantly higher than that after the expedition (0.82 ± 0.08 mg/l, *p* < 0.05) and lower vs the control group (1.10 ± 0.21 mg/l, *p* < 0.05). The copper content in the serum of the after-wintering expedition (0.62 ± 0.10 mg/l) was compared with the baseline data (1.11 ± 0.07 mg/l, *p* < 0.05) and controls (1.19 ± 0.17 mg/l, *p* < 0.05). The content of zinc in the serum, on the contrary, increased after the expedition (pre 0.88 ± 0.09 mg/l, in the control −0.90 ± 0.21 mg/l, after −1.27 ± 0.31 mg/l).

Such changes in trace-element composition in the blood of winterers indicate some adjustment in the body that can contribute likely dysregulation and disadaptation disorders to the oxygen-transporting system in the human body during prolonged stays in the extreme conditions of Antarctica.

### The “ozone hole”

The territory of the Antarctic “Akademik Vernadsky” station refers to the area with a high risk of ultraviolet (UV)-induced ophthalmic pathology and pathology of the skin. The results of wintering eye examinations showed that under the influence of excess UV radiation exposure there are naturally evolving changes in the organ of vision—parenchymal, endothelial keratopathy, nuclear and posterior subcapsular facopathy, and maculopathy. The pathological conditions are subclinical or clinical manifestations of dystrophy of the cornea, and cataracts and macular degeneration were noted [39]. For the skin, especially the frontal part, there are persistent erythematous areas, reducing the elastic properties of the skin and its roughness. Unlike humans, Antarctic biota is well adapted to the harsh effects of ultraviolet radiation. Retreaded properties are due to the specific peculiarities of the metabolism, and the presence of the producers increases the stability of the negative effects of UV. Bioactive substances studied in the Antarctic are already widely used in cosmetology and clinical practice (e.g., melatonin). Further studies focused on individuals, who lack the specific mechanisms to complete the adaptation to the Antarctic conditions, and the studies of the effect of the natural UV spectrum in an open “ozone hole” and the search and validation of the biological markers for the anticipation of disorders of psycho-physiological functions are still at the stage of data acquisition and analysis of monitoring surveys.

### Photoperiodicity, desynchronosis

Another important environmental factor is the monochromeity of the Antarctic environment, and the changing photoperiodicity downward light time causes certain disorders of the mental and emotional status of the person (affective disorder in the winter and there are outside Antarctica). The influence of the changed conditions of the biorhythmological factors (inversion season photoperiodism, monochrome, time zone) of the Antarctic station crew found significant shifts in the circadian architectonic structure body temperature indicators (a proxy for changes in the humoral regulation of biorhythm). Curving circadian rhythms were considered as representative characteristics of other functional systems. It was revealed that the greatest adjustment to the circadian architectonic structure body functions occurs in unusual photoperiodic periods (winter and summer) and the initial phase of adaptation to the conditions of the Antarctic. In the Antarctic station, the highest number of cases of violations of normal sleep, symptoms of headache, and worsening of mood and well-being was recorded during the Antarctic winter. At this time, the listed symptoms were observed in almost all the members of the team (95 %), resulting in increased risk of disorders of psycho-emotional stability and relationships in a small team. During the other seasons of the year, these syndromic and pathologic manifestations were observed in only half of the wintering. Similar symptoms in humans during the winter in the Antarctic, which were called “Antarctic syndrome,” were reported at many Antarctic stations in other countries [10–12].

*Insomnia and headache* complaints, reported only at the South Pole station, were likely associated with the increased elevation at that site, although they could be attributable to psychological stress from the isolated environment. Although the majority of cases could not be prevented with current screening, we suggest several changes to the current concept of operations that may decrease medical utilization and provide significant improvements to health care delivery on the ice [40].

The seasonal variation in mood and behavior was examined in 87 American men and women who spent the 1991 austral winter at three different research stations in Antarctica [41]. The South Pole station (90° S) crew reported a significant decline in tension/anxiety, depression, anger, confusion, and fatigue from March to August, followed by a significant increase in tension/anxiety and fatigue and a significant decline in vigor from August to October. The McMurdo station (78° 51′ S) crew also reported a significant decline in tension/anxiety from March to July and a significant increase in tension/anxiety from July to August. In contrast, the Palmer station (64° 46′ S) crew experienced no significant changes in any mood subscale from May to October. The non-linear pattern of change in mood suggests that the adaptation to prolonged isolation and confinement in an extreme environment occurs in two or three stages [41, 42].

*Gender aspects* in sleep quality have been studied in the Neumayer Antarctic stations [43]. Thus, overwinterings at the stations Neumayer II and III are associated with significant changes in sleep patterns, with dependences from overwintering time and local sunshine radiation. Gender appears to be an influence, as women showed a declining sleep quality, despite that their physical activity remained unchanged, suggesting other causes such as a higher susceptibility to psycho-social stress and changes in environmental circadian rhythm during long-term isolation in Antarctica [43].

Palinkas et al. [44] examined the physiological and psychological status of men and women who spent the summer (*n* = 100) and/or winter (*n* = 85) seasons in Antarctica at the McMurdo (latitude 78.48 S, elevation 12 m) and South Pole (latitude 90 S, elevation 3880 m) stations to determine whether there were any significant differences by severity of the stations’ physical environment. South Pole residents had a lower body mass index (*p* < 0.05) and body temperature (*p* < 0.01) and higher levels of plasma norepinephrine (*p* < 0.05) in the summer and winter than McMurdo residents. Upon deployment from the USA and during the summer, the South Pole residents experienced significantly higher thyroid hormone values (free and total T(3) and T(4)) (*p* < 0.01) than the McMurdo residents; in summer, they also experienced lower levels of triglycerides (*p* < 0.01), cortisol (*p* < 0.05), and LDL (*p* < 0.05). In winter, the South Pole residents also experienced a 39 % decrease in serum TSH compared with a 31.9 % increase in McMurdo residents (*p* < 0.05). The South Pole residents also were significantly more accurate (*p* < 0.05) and efficient (*p* < 0.01) in the performance of complex cognitive tasks in the summer and winter. Higher thyroid hormone levels, combined with lower BMI and body temperature, may reflect increased metabolic and physiological responses to colder temperatures and/or higher altitude at the South Pole with no apparent adverse effect on mood and cognition [44].

The increase in TSH, and its association with mood, is consistent with the polar T3 syndrome, while the absence of changes in free triiodothyronine and thyroxine may reflect characteristics of the environment or racial/ethnic differences in psycho-physiological or socio-cultural adaptation to circumpolar environments [45].

The “Antarctic syndrome” phenomena could be accompanied by autonomic dysfunction, which are characterized by hypotonic or hypertonic reactions, unstable heart rate, temporary rearrangement indicators of the heart electrical activity. The results of monitoring blood flow parameters in wintering during the year showed that the background of the individual fluctuations in heart rate, electrical activity of the heart, and blood pressure were overall a downward trend in pulse pressure and the appearance of signs of increasing strain on the heart. The characteristic symptoms of the psycho-physiological state during hibernation associated with the so-called desynchronization disorders [46] are the occurrence of thyroid dysfunction, changes in circulation modes of melatonin in the body, etc. Such developments are a testament for desynchronization disorders and require the development of effective routes for correction.

**Fig. 4:**
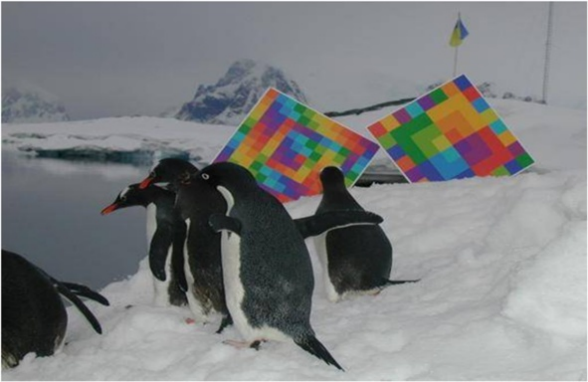
Color tables in the monochrome environment

It is noticed that the man in Antarctic monochromatic conditions has a deficit of color perception, which may adversely affect the psycho-emotional status. This state can be compensated briefly in the Antarctic by the rare effects of various types of auroras from the refraction of sunlight, which are observed mainly at sunset or sunrise. Colorful pictures of the sky that legitimately cause emotional reactions in wintering are subject to a kind of “hunting” for the original views of the environment and remembered for a long time. A distinctive feature of the Antarctic landscape is the almost complete absence of color in the green spectrum. The problem changes the psycho-emotional state of a person with long-term isolation in a small team and is relevant for the vast majority of the Antarctic stations. To solve this problem, we conducted studies of the human rearrangement color preferences in the Antarctic and the possibility of correcting his psycho-physiological state via the adaptive biological control method of the polychrome. Color effects on human pilot studies were carried out and in the Antarctic for the first time [46]. The polychrome-adaptive method is feasible and can be used for medical and preventive purposes in the monochromeity of the Antarctic environment and for biological control, which is the youngest branch of psychological correction with the help of colors and attracts simplicity, the lack of direct contact with the body, and the impact of efficiency. The technology is a non-invasive correction of the psycho-physiological status and is based on the author’s invention by Madjar [47]. It is based on the impact of dynamic color tables by individual sensory perception. The dynamics of the color composition is based on the harmonization of color triads and executed in a table and screen hardware and software versions. A general view of the color tables in the monochrome environment is demonstrated on Fig. 4.

In order to determine the prevalence of the conditions in the Antarctic syndromic manifestations in humans, parallel studies were conducted at the Polish research station, which is located in a different region of the coastal zone (62° 09′34″ S; 58° 28′15″ W) [42]. In the initial stages of the stay in the Antarctic, crew members of both stations observed deterioration in the mental and emotional states. During the Antarctic winter and Antarctic summer, desynchronosis syndrome disorders provoked various diseases and required the use of therapeutic interventions. In addition, the manifestation of the “Antarctic syndrome” is essential in the formation of symptoms of chronic stress impairing oxidant-pro-oxidant system and the development of a number of maladaptive disorder*s*. Physiotherapy techniques (pneumopressing massage therapy, local and general thermal and electromagnetic wave treatments) and pharmacological agents (melatonin, etc.) did not provide full-effect correction. Antarctic scientists in other countries also studied for years [48–50] and failed to achieve the desired success in solving the problem of correction of a psycho-physiological state of a person in Antarctica. The application of dynamic color images in tables allowed in 84 % of wintering normalizes the parameters of blood circulation, stabilizes the physiological characteristics of the observed responses of relaxation, and falling asleep at the end of the session [48]. During the study, a single case of absence of a relaxing effect has been identified that may be due not only to the psycho-emotional state before the session but also to the body’s sensitivity to individual extreme activity of the regional environmental factors (weather, helio-geophysical factors). Under the normal psycho-physiological state of wintering perception of dynamic color tracks, as a rule, caused relaxation effects during the winter.

### “Sterile” environment

Under the conditions of prolonged isolation of small communities in Antarctica it was formed a special kind of balanced microbial environment. In this case, the immune system of the body is experiencing a kind of “detraining” in relation to the commonplace appearance of microbial diversity [51, 52]. This is manifested by decreased activity of the protective properties of parts of the immune system without marked changes in the structural and morphological composition of white blood cells. At the same time, signs of chronic stress and adaptation disorders additionally help to reduce the body’s immune resistance, probably due to changes in the system of prooxidant-antioxidant homeostasis (the development of oxidative stress). As a result, after long-term isolation in a large part of the wintering, at the first contact with the introduced microflora (for example, when dealing with other people), there may be generalized reactions by type of respiratory disease. *Prevention* of such reactions is quite a challenge, since traditional methods of prevention demonstrate low efficacy. Here, apparently, it is necessary to focus more on relevant biomarkers that will assess the level of threat, and opportunities to strengthen immune resistance due to immune modulatory agents.

Antarctic microbial biogeography and atmospheric circulation in the south-polar region provide unique opportunity for comparative studies of atmospheric ecology of Antarctica and in the Arctic that need for standardized sampling and analysis protocols. These aerobiology metadata might be used for over a range of applications, including pollen dispersal (and allergy susceptibility), species invasions, spread of diseases, and air pollution [53].

Thus, at the station “Akademik Vernadsky,” psychrotolerant bacteria which use obligately methane were found in the moss samples and in soil-vegetation samples in the island part in Antarctica during the VII Ukrainian expedition (2003) [51].

The development of effective, scalable restoration tools and approaches will inevitably be complicated by its broad multi-disciplinary nature. Therefore, whatever the future direction, if ecological restoration is to result in reliable applied science, then strong collaboration will be required among ecological, economic, and social experts, as well as with private and public stakeholders, to encompass a diverse array of fields into a transdisciplinary co-designed approach [54].

We also suggest novel insights for a number of beneficial applications of research in host-microbiome interaction, probiotics, and personalized dietology for advances in development of novel probiotic-based treatments and personalized diets, promotion of health based on smart patient profiling with relevant gut microbiome data [16]. Thus, the mammalian gut microbiome is involved in controlling the circadian rhythm of its host. Metabolites produced by gut microbes in mice can affect the animals’ circadian rhythm and metabolism [55].

“Sterile” environment is an opportunity for many unbiased research of microbiome that is not possible out of Antarctica.

### Intriguing directions and hypotheses for vascular dysregulation, pain, and carcinogenesis

Many findings on Antarctica [26, 38] in regard to the vascular regulation and adaptation to stress tolerance have clear links with research on vasospastic phenomena like Flammer syndrome [17] and pain with prospects to further progress in the study of ischemia and stress for affecting for central sensitization and diverse regulatory mechanisms.

For example, clinical investigators to explain the pathophysiology of headache (migraine) put forward a wide range of hypotheses of its occurrence:

- The restriction of the lumen of arterioles and reduction of cerebral blood flow
- Reduction cerebrovascular reactivity in relation to carbon dioxide, uneven vasodilation
- Neuro-vascular disorders of the central nervous system, which serve as a trigger changes in vasomotor regulation
- System violation of metabolic regulation with seizures secondary to intravascular changes associated with impaired serotonin metabolism

In addition, recent data demonstrate the usefulness of the study of lipid peroxidation as a pathogenic factor in the development of migraine, as the activation of lipid peroxidation—one of the urgent adaptation mechanisms of the body to the action of pain stress [56]. In addition, in the pathogenesis of migraine as a “secondary” cell unit observed violations of energy. The role of the oxidative pathway in migraine is still uncertain. Interesting evidence emerged for TBARS and SOD and concerning the possible role of diet in the control of NOx levels [56].

Ischemia might be a source for a potential marker for carcinogenesis [14, 57]. Considering the complexity of vasospastic phenomenon, pain, and stress response patterns, taken together molecular and genetic aspects of the origin of adaptation, oxidative pathway, immune resistance due to immune modulatory agents might support panel of biomarkers for screening chemicals for carcinogenic potential.

Novel integrated concept for pain management in the consolidated paradigm of PPPM [15] should be implemented in the highest levels of multidisciplinarity and benefits from excellent competencies of all medical fields and complex technological instruments (including hybrid technologies). Gut-brain axis altered in “sterile” environment might be hypothesized as strong regulating mechanism [16]. Genomics studies of pain will provide fascinating data to complement the integrative vision [58, 59].

Results might help to stratify patients with diseases such as cancer, NDD, metabolic syndrome, pain, psychological disorders, and “emotional burnout” syndrome for personalized therapies, improving the therapeutic outcome of major chronic diseases for individual patients.

### Study limitations

The main limitation of the study was number of participants in the annual team, who were relatively healthy males, which limited possibilities to apply a broad spectrum of diagnostic methods on site (like functional magnetic resonance imaging (fMRI), laboratory tests), local medical monitoring, and telemedicine methods for patients’ records in the one standard.

### Consolidation of the PPPM concept

The results of the retrospective study and multi-domain observations in the Antarctic are potentially applicable in the scope of the predictive, preventive, personalized medicine for the following:

### Predictive medical approach

It is recommended to promote the concepts of patient stratification according to the obtained data in predictive programs for personalized therapies and prevention chronic diseases; to organize educational programs for radiologists to create screening network with unified procedures in strong inter-observer agreement to evaluate participants prior to the expedition to predict unfavorable outcomes at the station; and compare disease course/outcome at the station vs in Ukraine and their follow-up examination in Ukraine during the years.

Results of current and upcoming studies might justify the appropriate conclusion for recommendations and guideline development that may be done for the EU/Ukrainian society for improving health care via preparing international projects.

### Preventive medical approach

The research results indicated the importance of disease prevention in winterers as follows: medical screening, appropriate use of therapeutic and preventive tools, special education, and training station operators to prevent individual/social dysadaptation, diseases at the station and after expedition with appropriate recommendations.

Monitoring of individual health indicators of Antarctic winterers allows control of their functional state and the very early stages to prevent the development of dysadaptative disorders and disease by applying biotechnologies for appropriate correction.

On the other hand, the monitoring of biomedical research performance with the parallel registration of the dynamics of extreme environmental factors in the environmental conditions of Antarctica allow new evaluation criteria to find out preventive values and identify additional mechanisms of development of many pathological conditions of unknown etiology.

With such a variety of problems of preventive measures hypotheses become apparent. Therefore, their solution is necessary to develop research in the direction of preventive medicine and physiology with medical observation opportunities environmentally clean conditions in Antarctica. Ultimately, the effectiveness of medicine will be assessed at the level of health protection and health operators.

### Personalized medical approach

Gathering large volume of personal profile data including genetic/molecular profiles and their alteration in regard to individual responses to stress, environment, and diseases will ensure a personalized approach.

## Conclusions

Thus, the preliminary results of the retrospective study and our own observations of fundamental physiological mechanisms of the influence on an organism of environmental factors in the absence of man-made origin factors make it possible to identify new ways for the development of original technologies of disease prevention and the preservation of human performance in extreme conditions and in conditions of negative impact of the variability of ecological environment.

### Outlook and expert recommendations

To establish collaboration between National Antarctic Scientific Center of Ministry of Education and the reputed international medical societies as EPMA is preparing conjoined projects to promote the paradigm of PPPM and utilize unique opportunities in the Antarctic. Creating relevant international EU consortia to run large multi-disciplinary prospective (preferred RCT) studies and long-lasting follow-up of the groups with extensive and standard medical records.

Research plan for the prospective studies, and those that have been set up so far, include as follows:

- Predictive biomarkers for stress and dysadaptation prognosis and prevention
- Genomic studies to detect altering genetic data including advanced omics techniques like glycome and activome [60] and study the correlation with epigenetic with microbiome and metabolome
- “unbiased” research of the microbiome of a wide microbal spectrum (including anaerobes, fungi and viruses) of the gut and also on the distant sites (skin, gut, nose, saliva, pharynx, urethral, circadian rhythms) with organization on-site monitoring
- Studies on vascular dysregulation predisposition parameters and Flammer syndrome questionnaire data for winterers
- Study on pain, psyche, burnout in the concept of dysregulation and dysadaptation
- Study on sport and physical regimes for sustainable well-being
- Studies on allergy, asthma, immune system, and comorbid conditions
- Dietary studies, use of pre-/probiotics, and food supplements
- Studies on ecology and environmental-health associations
- Imaging records and telemedicine development, ultrasound, fMRI, PET studies, and studies to evaluate radiomics-metabolomics-genomics correlation
- Suggest and discuss innovative tools for diagnosis, screening, treatment prevention, and developing innovative techniques on EEG, ECG, Doppler, capillaroscopy, nanosensors, imaging, etc., data to implement to the software and mobile gadgets
- Study and promote the “value-based approach” [18] for the results implementation

## Abbreviations

CNS: central neural system
ECG: electrocardiography
EEG: electroencephaolography
ENT: otolaryngologists
fMRI: functional magnetic resonance imaging
IAPS: International Affective Picture System
NASC: National Antarctic Scientific Center
PET: positron emission tomography
PPPM: predictive, preventive, personalized medicine
CI: cardiac index
SOD: superoxide dismutase
SP: pre-excitation syndrome
STC: Scientific and Technical Council
SVR: systemic vascular resistance
TBARS: thiobarbituric acid reactive substances
