# Erratum to: Antarctica challenges the new horizons in predictive, preventive, personalized medicine: preliminary results and attractive hypotheses for multi-disciplinary prospective studies in the Ukrainian “Akademik Vernadsky” station

**DOI:** 10.1186/s13167-016-0067-1

**Published:** 2016-07-18

**Authors:** Yevhen V. Moiseyenko, Viktor I. Sukhorukov, Georgiy Yu Pyshnov, Iryna M. Mankovska, Kateryna V. Rozova, Olena A. Miroshnychenko, Olena E. Kovalevska, Stefan-Arpad Y. Madjar, Rostyslav V. Bubnov, Anatoliy O. Gorbach, Kostiantyn M. Danylenko, Olga I. Moiseyenko

**Affiliations:** 1 National Antarctic Scientific Center of Ministry of Education of Ukraine, 16, Taras Shevchenko Boulevard, Kyiv, 01601 Ukraine; 2 Bogomoletz Institute of Physiology, National Academy of Sciences of Ukraine, 4, Bogomoletz str., Kyiv, 01024 Ukraine; 3 Institute of Neurology, Psychiatry and Narcology of the National Academy of Medical Sciences of Ukraine, 46, Akademika Pavlova str., Kharkiv, 61068 Ukraine; 4 Institute for Occupational Health of National Academy of Medical Sciences of Ukraine, Saksaganskogo str., 75, Kyiv, 01033 Ukraine; 5 Zhytomyr Ivan Franko State University, 40, Velyka Berdychivska Str., Zhytomyr, 10008 Ukraine; 6 G.S. Kostyuk Institute of Psychology of the National Academy of Pedagogical Sciences of Ukraine, 2, Pankivska str., Kyiv, 01033 Ukraine; 7 Budapest University of Technology and Economics, Budapest, Hungary; 8 Clinical Hospital ‘Pheophania’ of State Management of Affairs Department, 21, Zabolotny str., Kyiv, 03680 Ukraine; 9 ZabolotnyInstitute of Microbiology and Virology, National Academy of Sciences of Ukraine, 154, Zabolotny Str., Kyiv, 03680 Ukraine; 10 Ukrainian Academy of Informatics, Kyiv, Ukraine; 11 National Scientific Center ‘Mykola Strazhesko Institute of Cardiology’ of National Academy of Medical Sciences of Ukraine, 5, Narodnoho Opolchennya str., Kyiv, Ukraine

## Erratum

After publication of the original article [1] it was brought to our attention that incorrect data was given in the second paragraph of the ‘Methods’ section.

The original text reads as:

In addition, their duration in the Antarctic is much more powerful and higher than that registered in Ukraine. Subzero temperatures in the winter rarely exceed 300 °C and in summer during a very short period (only during the day) can rise over 0 °C. The average annual temperature in the region of the station is 40 °C, compared to +90 °C in Ukraine.

However, the corrected version should now read as follows:

In addition, their duration in the Antarctic is much more powerful and higher than that registered in Ukraine. Subzero temperatures in the winter rarely exceed -30 °C and in summer during a very short period (only during the day) can rise over 0 °C. The average annual temperature in the region of the station is -4 °C, compared to +9 °C in Ukraine.

This has been corrected on the BMC website.
